# Targets and self-management for the control of blood pressure in stroke and at risk groups (TASMIN-SR): protocol for a randomised controlled trial

**DOI:** 10.1186/1471-2261-13-21

**Published:** 2013-03-23

**Authors:** Claire O’Brien, Emma P Bray, Stirling Bryan, Sheila M Greenfield, M Sayeed Haque, FD Richard Hobbs, Miren I Jones, Sue Jowett, Billingsley Kaambwa, Paul Little, Jonathan Mant, Cristina Penaloza, Claire Schwartz, Helen Shackleford, Jinu Varghese, Bryan Williams, Richard J McManus

**Affiliations:** 1 Primary Care Clinical Sciences, NIHR School for Primary Care Research, University of Birmingham, Edgbaston, Birmingham, B15 2TT, UK; 2 Centre for Clinical Epidemiology and Evaluation, Vancouver Coastal Health Research Institute, and School of Population and Public Health, University of British Columbia, Vancouver, BC, Canada; 3 Primary Care Health Sciences, NIHR School for Primary Care Research, University of Oxford, Radcliffe Observatory Quarter, Woodstock Road, Oxford, OX2 6GG, UK; 4 Health Economics Unit, School of Health and Population Sciences, University of Birmingham, Edgbaston, Birmingham, B15 2TT, UK; 5 School of Medicine, University of Southampton, University Road, Southampton, SO17 1BJ, UK; 6 Primary Care Unit, Institute of Public Health, University of Cambridge, Forvie Site, Robinson Way, Cambridge, Cambridgeshire, CB1 8RN, UK; 7 Institute of Cardiovascular Sciences, University College London, 170 Tottenham Court Road, London, W1T 7HA, UK

**Keywords:** Hypertension, Self-management, Stroke, Diabetes, Coronary heart disease, Chronic kidney disease, Primary care

## Abstract

**Background:**

Self-monitoring of hypertension with self-titration of antihypertensives (self-management) results in lower systolic blood pressure for at least one year. However, few people in high risk groups have been evaluated to date and previous work suggests a smaller effect size in these groups. This trial therefore aims to assess the added value of self-management in high risk groups over and above usual care.

**Methods/Design:**

The targets and self-management for the control of blood pressure in stroke and at risk groups (TASMIN-SR) trial will be a pragmatic primary care based, unblinded, randomised controlled trial of self-management of blood pressure (BP) compared to usual care. Eligible patients will have a history of stroke, coronary heart disease, diabetes or chronic kidney disease and will be recruited from primary care. Participants will be individually randomised to either usual care or self-management. The primary outcome of the trial will be difference in office SBP between intervention and control groups at 12 months adjusted for baseline SBP and covariates. 540 patients will be sufficient to detect a difference in SBP between self-management and usual care of 5 mmHg with 90% power. Secondary outcomes will include self-efficacy, lifestyle behaviours, health-related quality of life and adverse events. An economic analysis will consider both within trial costs and a model extrapolating the results thereafter. A qualitative analysis will gain insights into patients’ views, experiences and decision making processes.

**Discussion:**

The results of the trial will be directly applicable to primary care in the UK. If successful, self-management of blood pressure in people with stroke and other high risk conditions would be applicable to many hundreds of thousands of individuals in the UK and beyond.

**Trial Registration:**

ISRCTN87171227

## Background

The potential benefit from optimal blood pressure (BP) lowering in patients at high cardiovascular risk following stroke or TIA, coronary heart disease or with diabetes or CKD is large. The PROGRESS trial demonstrated that blood pressure lowering is beneficial in reducing risk of stroke amongst both hypertensive and non-hypertensive individuals with a history of stroke or TIA [1,2]. For people with coronary heart disease, blood pressure lowering has the same risk reduction as in those without coronary heart disease however the higher absolute risk in CHD means that for a given blood pressure reduction the absolute benefits are greater [3]. The Hypertension Optimal Treatment trial showed no difference in outcome for diastolic blood pressure targets below 90mmHg, apart from in people with diabetes for whom the 80mmHg target group did better. The blood pressure trialists collaborative have shown similar relative risk reductions from blood pressure lowering in diabetes compared to other groups, again with higher absolute risk reductions. In subgroup analyses of the HOPE study, people with chronic kidney disease (CKD) received equivalent benefit from ramipril as those without kidney disease [4].

Guidelines for the various at risk groups vary in terms of recommendations for blood pressure lowering. The National Clinical Guideline for stroke [5] and the British Hypertension Society (BHS) [6] recommend that unless there is bilateral carotid artery stenosis, the target blood pressure for secondary prevention of stroke and TIA should be 130/80mmHg. NICE guidelines for diabetes, suggest a lower blood pressure target than recommended for essential hypertension of 140/80mmHg (130/75mmHg in cases of proteinuria). For coronary heart disease, standard blood pressure targets are recommended (≤ 140/90 mmHg), and for chronic kidney disease NICE also recommend a target of 140/90 mmHg, unless there is accompanying diabetes or proteinuria (ACR > 70 mg/mmol) in which case the target drops to 130/80 mmHg. The BHS guidelines however, suggest a target of <130/80 mmHg for stroke/ TIA, diabetes, CKD3 (without proteinuria), CHD and MI allowing uniformity across the range of high risk groups [6].

Data from national and international surveys suggest that blood pressure control is sub-optimal [7]. Novel interventions are therefore needed to improve this and as most blood pressure management is undertaken in primary care, where hypertension is the commonest long term condition seen by GPs, it is appropriate that interventions are delivered in this setting. The TASMINH2 trial [8,9] found that self-management of hypertension resulted in significantly lower (5.4mmHg) systolic blood pressure after one year compared to usual care. However, the study included few people in high risk groups such as diabetes or CKD, in whom the effect size appeared to be smaller and included telemetry which is not available in daily practice in the NHS.

Self-management can encompass a wide range of behaviours in addition to medication titration and monitoring of symptoms, such as an individual’s ability to manage physical, psychosocial and lifestyle behaviours related to chronic illness [10]. Self-efficacy, which is a person’s confidence to be able to carry out behaviours to achieve a desired goal, has been found to be the strongest predictor of a person’s ability to change risky health behaviours by taking action, and an important characteristic for successful self-management [11]. It is unclear what the relationship is between self-monitoring of blood pressure, self-efficacy and health behaviour modification; it is possible that the self-monitoring aspect provides feedback to the individual about their blood pressure of which they would otherwise be unaware. This in turn may promote self-management of health behaviours in those with higher levels of self-efficacy. These behavioural aspects require further study.

Therefore, the aim of this trial is to determine whether the benefits from blood pressure lowering observed in the TASMINH2 trial will also be observed in a population of people at high cardiovascular risk without using telemetry and to assess further the mechanism behind any change in blood pressure observed. The TASMIN-SR trial sets out to investigate whether self-management is effective and cost effective in people with stroke and other high risk conditions.

## Methods/Design

### Study aims, research questions, and outcomes

The primary aim of TASMIN-SR is to compare self-management with usual care in the control of hypertension in patients with stroke and other at-risk conditions.

The trial has four main research questions:

1. Does self-management of blood pressure result in better control of blood pressure in people with stroke and other at-risk conditions compared to usual care?

2. Is self-management of blood pressure in people with stroke and other at-risk conditions achievable in routine practice and is it acceptable to patients?

3. What is the relationship between self-management of blood pressure, self-efficacy, lifestyle behaviours, patients’ attitude to health and health care and use of other self-care strategies in people with stroke and other at-risk conditions?

4. Is self-management of blood pressure in people with stroke and other at-risk conditions cost effective?

The primary outcome of the trial will be the difference in office systolic blood pressure (mmHg) at 12-month follow-up between intervention and control adjusted for baseline blood pressure and co-variates. Secondary outcomes (also adjusted for baseline and co-variates) will include:

–  Difference in office SBP at 6-month follow-up between intervention and control

–  Difference in office DBP at 6 and 12-month follow-up between intervention and control

–  Percentage time in target BP range

–  Difference in pulse rate

–  Difference in self-management self-efficacy

–  Difference in lifestyle behaviours

–  Difference in health-related quality of life

–  Difference in BP measurement preference

–  Difference in anxiety

–  Difference in attitudes to health and healthcare

–  Difference in use of other self-management strategies

–  Reasons for non-participation

–  Adverse events (including cardiovascular events and death)

–  In addition there will be a qualitative analysis and health economic modelling.

### Study design and setting

TASMIN-SR is a pragmatic, primary care based, unblinded, randomised controlled trial (with embedded economic and qualitative analyses) of self-management of BP consisting of self-monitoring with self-titration of anti-hypertensive medication in people with stroke and other at-risk conditions.

### Ethical considerations

Ethical approval has been obtained from North West – Greater Manchester East ethics committee (reference: 10/H1013/60). Site specific R&D approval will be obtained from the relevant Primary Care Trusts.

### Trial interventions

Usual care will consist of the participant seeing their General Practitioner (GP) and/or nurse for routine BP measurement and/or adjustment of medication at the discretion of the health professional.

Self-management will consist of self-monitoring of BP with self-titration of medication following a predetermined 3-step plan, dependant on the self-monitored BP readings.

#### Blood pressure self-monitoring

Participants will be trained to self-monitor BP using an automated sphygmomanometer. Patients will self-monitor BP for the first week of each month of the study, and will take measurements in the morning. Two seated BP readings will be taken, with a five-minute rest period between them. The second of these readings will be used to determine if medication requires altering. Participants will be provided with a guideline that contains simple colour coded instructions. Very high or very low readings that persist when a third reading is taken five minutes after the second reading will require the participant to contact their practice for advice and potentially will need checking. Four or more above target readings in two consecutive weeks of measurement will require a change in medication. Readings within target range will simply require further monitoring the following month.

#### Target blood pressure

Blood pressure targets will be based on The British Hypertension Society guidelines [6] and Joint British Societies Guidelines [12] that suggest that the BP for patients with stroke/TIA, diabetes (in the absence of proteinuria), CKD, CHD, and MI should be <130/80 mmHg. The BHS suggest that for home monitoring this target should be adjusted by 10/5 mmHg, resulting in a target of <120/75 mmHg [6].

#### Communication of home readings

Participants will complete a simple form each month to record their daily BP readings and colour coding. These forms will be used to determine any action that is required at the end of the measurement week, including whether a medication change is required. The form will be printed on three-part non-carbon copy paper to allow one copy to be kept by the patient, one returned to the research team, and one posted to the GP should a medication change be required. Reply paid envelopes will be provided for this purpose. At follow-up, data from participants’ BP machines will be uploaded onto a database so that the research team has an electronic copy.

#### Self-titration of medication

Each intervention patient will be given an individually tailored three-step management plan through which to adjust medication according to measured BP. Each step will represent a single medication change (additional medication or increased dose) that will be made following two consecutive months of raised readings. Medication choice will remain at the discretion of the GP who will be provided with an algorithm summarising the national clinical guidelines for advice on hypertension. If patients use all three steps of their management plan they will return to their GP and an additional two-step plan will be devised. This will not be until at least eight months into the trial, assuming no very high or very low readings, so a three-step plan should be sufficient. Any additional monitoring (for instance blood tests or urinalysis) will be the responsibility, and at the discretion, of the GP.

### Non-participation

Included with the letter of invitation to take part in the trial, will be a form for people to voluntarily return should they wish to decline the invitation. This will ask for basic demographic details as well as their reasons for wishing to decline.

### Study population

The study population will comprise people with stroke/TIA, diabetes, CKD3, CABG, MI or angina, with poorly controlled hypertension managed in primary care. Eligibility criteria will be age above 35, have had a diagnosis of stroke/TIA, diabetes, CKD3, MI, angina, or CABG, and clinic blood pressure greater than 130/80. Exclusion criteria will be inability to self-monitor (such as dementia or score of >10 on the short orientation memory concentration test), postural hypotension (systolic BP drop > 20 mmHg), prescribed more than three anti-hypertensive medications, taking part in a current BP study or previously having taken part in TASMINH2 [8], terminal disease, pregnant, BP not managed by GP, and acute cardiovascular event in the previous three months.

Eligible patients will be identified from general practices via the UK Primary Care Research Network. Trained practice nurses will identify potentially eligible patients by searching practice-based registers for patients having a Read Code of stroke/TIA, diabetes, CKD3, Angina, CABG, or MI and whose last systolic BP measurement was greater than 145 mmHg (BP readings are often lower when measured by research teams, so a higher BP at invitation increases the likelihood of BP readings falling within the suitable range [13].) GPs will be asked to check the generated lists and remove patients who have a terminal illness, are pregnant, or who are thought to be unsuitable for the study.

Participants who withdraw will not be replaced, but asked if they are prepared to continue to attend follow-up clinics.

### Randomisation

Patients will be randomised to either usual care or self-management using an internet based system with telephone backup. Minimisation will be used to take into account practice, gender, age, high risk group (CVD, diabetes, CKD3, CHD) and baseline BP.

### Study clinics and flow through study

At baseline, all patients will attend a clinic at which the study will be explained, informed consent gained, height, weight, and BP measurements taken, and questionnaires regarding demographics, past medical history, BP measurement method preference, use of self-management strategies, attitudes to health and healthcare completed, and baseline economic data collected (Table 1). Measurement of blood pressure will use a validated automated electronic sphygmomanometer (BP TRU BPM 200; BP TRU Medical Devices; Coquitlam, BC, Canada) [14]. After five minutes of rest, six seated blood pressure readings will be taken at 1-minute intervals, of which the mean of the 2^nd^ and 3^rd^ reading will comprise the primary outcome. Patients will then be randomised to either usual care or self-management. All patients will be given a diary to assess daily lifestyle behaviours and self-management self-efficacy which they will be asked to complete everyday for one week starting the first Monday of the month after their baseline appointment. Patients randomised to usual care will be asked to book an appointment for a routine blood pressure check and medication review with the study GP. Patients randomised to self-management will be asked to make an appointment with the research team for a training session on how to monitor their BP. Participants will be asked to practice at home for a week before returning for a second training session covering the self-titration aspect of the intervention. If necessary, a third training session will be offered for additional support. Following successful completion of the training, patients will be asked to make an appointment with the study GP at their practice for a routine BP check and to devise a three step titration plan for any potential medication changes. Patients who are unable to complete all aspects of the training will be given the option to self-monitor without self-titration of medications.

**Table 1:** Data collection throughout the trial

**Baseline only:**	
1	Demographic questions: including age, race, marital status, occupation, and education
2	Duration of hypertension
3	Past medical history
4	Contraindications to anti-hypertensives
5	Short orientation memory test [15]
6	Height
7	Joint pain questionnaire [16]
Baseline and follow-up:	
1	New medical history (in last 6/12 months)
2	Blood pressure (sitting plus standing at baseline)
3	Current anti-hypertensive medications
4	Weight
5	Symptom section of the IPQ [17]
6	Partners in health scale [18]
7	Short-form of the State-Trait Anxiety Inventory [19]
8	EQ-5D [20]
9	Use of complementary and alternative medicine and self-tests [21]
10	BP measurement preference
11	Attitudes to health and healthcare [22]
Lifestyle diaries	
1	Simple lifestyle indicator questionnaire (SLIQ) [23]
2	The dietary quality score [24]
3	Self-efficacy (adapted diabetes self-efficacy scale) [25]

**Figure 1:**
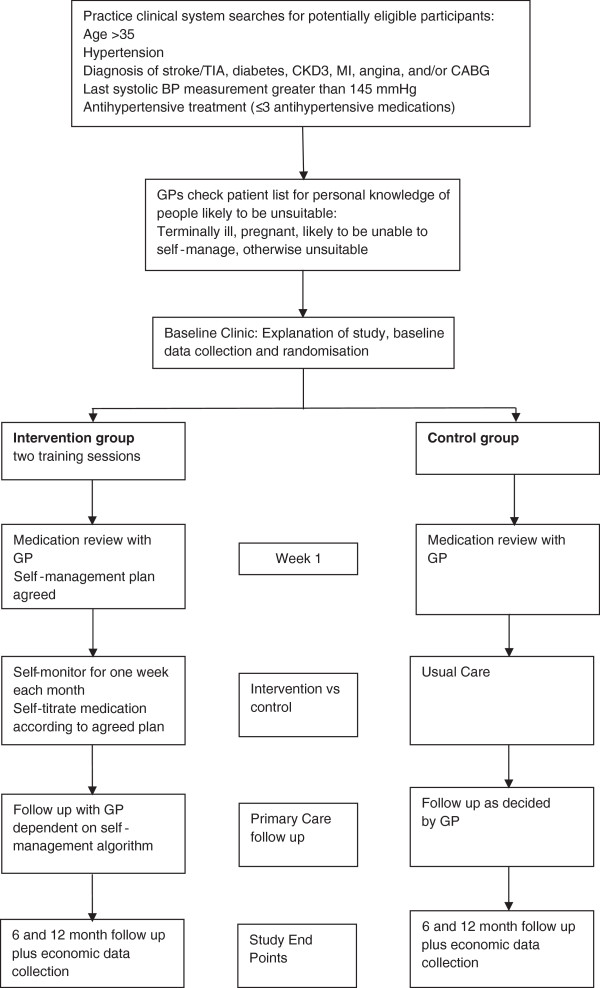
Flow through the trial.

Patients will be asked to attend two follow-up clinics at 6- and 12-months post-randomisation. Each clinic will be timetabled for no more than one hour, during which patients will have their BP and weight measured by the research team and will be asked to complete a questionnaire similar to the one completed at baseline. At the 12 month follow up, participants will also be given a blank postcard and asked to write a few sentences about their experience of the trial. Additionally, the research team will check that patients in the intervention arm are using the blood pressure monitors correctly. Flow through the trial is summarised in Figure 1.

### Sample size considerations

A sample size of 243 people per group is required for 90% power assuming a standard deviation of 17 mmHg and a difference of at least 5 mmHg between intervention and control groups. This represents a clinically significant decrease in BP and is in line with the reduction observed in TASMINH2 and would result in around 20% reduction in stroke risk and 10% coronary heart disease risk. Based on the follow-up in TASMINH and TASMINH2 self-monitoring trials, a 10% drop out rate during follow-up is assumed, meaning a sample of 270 per group will need to be randomised, a total of 540 patients altogether. Should the drop-out rate be higher than TASMINH2, for instance 20%, the study would have more than 85% power.

### Recruitment

Patients will be recruited over an eight month period. Based on practice-based pilot searches, it is estimated that in a practice with an average size of 6000 patients, 2.5% will be eligible for invitation. Previous experience from the TASMINH2 trial suggests that approximately 30% of invited patients will attend baseline clinics, and of these 50% will be eligible [8]. A minimum of 25 practices will be needed in order to recruit the required number of participants (approximately 22 patients per practice being randomised) but many more will be available if necessary.

### Statistical analysis

The primary analysis will include all available participants, i.e. all of those with complete data from follow-up, and will be performed at the end of the trial after all data has been collected. A mixed model analysis will be used to examine differences between intervention and control systolic BP at twelve months, adjusting for practice (as a random effect), baseline BP, gender, and high risk group. Planned sub group analyses will be of older vs younger (65 as threshold), males vs females, better controlled at baseline vs worse controlled at baseline (threshold 145 systolic), the different risk groups and deprivation. Sensitivity analyses will examine the potential effect of missing data. These will include multiple imputation, replacement of missing data by the most recent previous data or by the mean of the series. Any deviation from the original statistical plan will be described in the final report and publications.

### Economic analysis

The economic analysis will be in two parts. The first part is a cost-effectiveness analysis conducted alongside the randomised clinical trial (trial-based analysis). An economic evaluation will compare the strategy of self-management of blood pressure in at-risk patients to the strategy of usual care. Primary outcome will be expressed in terms of the cost per additional 1 mm Hg reduction in office SBP from baseline to 12 months. Use of utility-based outcomes (EQ-5D) will allow a secondary outcome to be the cost per quality-adjusted life year (QALY) gained over the same 12 months period. The results for both outcomes will be expressed in terms of incremental cost-effectiveness ratios (ICERs). NHS resource use will include hospital and GP consultations, medications, referrals, equipment and training. Intervention costs, including equipment and training, will be collected by the research team. All other resource use data will be collected from practice computer systems by the research team at follow up visits. Cost data will be derived from sources such as the British National Formulary (BNF), the National Schedule for Reference Costs and the Unit Costs of Health and Social Care (PSSRU) [26].

The second part will be a model-based cost-effectiveness analysis, building on the trial-based analysis and using published data on long-term outcomes and costs. The model will estimate the long-term cost-effectiveness of self-management of blood pressure in people with stroke and other at- risk conditions in terms of cost per QALY gained. The model type and structure will be informed by reviewing modelling studies which consider outcomes after stroke and other at-risk conditions. Experts within the team will advise on the final structure of the model. Costs to be included in the model will be for self-management (from the trial based analysis), hospital stays, readmissions and long-term care for stroke and other cardiovascular outcomes related to level of disability and discharge destination. Resource use will be determined from the trial and estimates from the literature. Unit costs will be collected from published sources (National Schedule for Reference Costs and the PSSRU) [26]. Outcomes will be in the form of survival and quality of life and will use data collected from the trial and literature on quality of life after stroke. The model will be run over remaining patient lifetime, with costs and benefits discounted at a rate of 3.5%. The analysis will be conducted from an NHS perspective. Extensive deterministic sensitivity analysis will be undertaken to assess the impact of changing the values of key parameters. For each important model parameter, we will determine a point estimate and construct a probability distribution around that estimate. Probabilistic sensitivity analyses will be conducted to deal with uncertainty in model parameters and cost-acceptability curves presented.

### Qualitative sub study

This part of the study aims to gain insight into patients’ decision making processes regarding whether to seek professional advice, whether to make a medication change, or any concerns they may have.

#### Open comments

Each month, on an open-comment section of the BP measurement record, intervention patients will be asked to write down their description of any action they took, whether it followed protocol or not, their decision making process, and their thoughts and feelings associated with the decision making. Similarly, the postcards participants will be given at the end of the study provide a further opportunity for open comment about the trial. This approach is useful for capturing aspects of a patient’s experience of a study or an intervention which may otherwise not be documented [27]. The open comments will be analysed by content analysis using both quantitative (e.g. number of times a word/phrase mentioned) and qualitative (e.g. examples of participants’ own words to reflect emerging themes) techniques. Concepts identified will be integrated into themes providing a structure for presentation of findings.

## Discussion

The results of the trial will be directly applicable to primary care in the UK. If successful, self-management of blood pressure in people with stroke and other high risk conditions would be applicable to many hundreds of thousands of individuals in the UK and beyond.

It is anticipated that the potential risks of this study are low and similar to those associated with usual care. Particular issues are potential increased anxiety when patients find excessively high or low blood pressure readings, or as a result of self-titration. The patient guideline will advise contact with the supervising physician or nurse for a blood pressure check and further management if required. Training of participants will cover repeated measurements in the case of high or low readings and a helpline will be available should participants or clinical staff require advice over and above that provided in the guideline. The study GP will have control over prescription of all medications within the study, and will make changes to prescriptions as required. Participants will be advised to attend their GPs should they experience an adverse event thought to be due to their participation in the trial.

## Competing interests

RJM has received equipment for research purposes from Omron and Lloyds Healthcare. FDRH has received limited research support in terms of blood pressure devices from Microlife and BpTRU. All other authors declare that they have no conflicts of interest.

## Authors’ contributions

RJM conceived the study and in collaboration with JM, SB and FDRH gained the funding. The protocol was developed by the trial investigators group comprising all the authors. The first draft of this paper was written by CO with RM and subsequently edited and approved by all co-authors. All authors read and approved the final manuscript.

## Article summary

This is the protocol for a randomised controlled trial comparing self-management of blood pressure (BP) with usual care in people with previous stroke, coronary heart disease, diabetes or chronic kidney disease.

The primary research question is: does self-management of blood pressure result in better control of blood pressure in people with stroke and other at-risk conditions compared to usual care?

## Pre-publication history

The pre-publication history for this paper can be accessed here:

http://www.biomedcentral.com/1471-2261/13/21/prepub
